# Supplementary material for: Development of Oxygen-Bridged Pyrazole-Based Structures as Cannabinoid Receptor 1 Ligands
Source: Molecules. 2019 Apr 27;24(9):1656. doi: 10.3390/molecules24091656 (PMC6539809; doi:10.3390/molecules24091656)
Supplement: Supplementary file 1 [file molecules-24-01656-s001.pdf]

Deepening the oxygen-bridged pyrazole-based structures as cannabinoid receptor 1 ligands

Table S1. Relative energy (E_{rel}, kcal/mol) and selected torsional angles (°) of conformers of compounds **1a-f**.

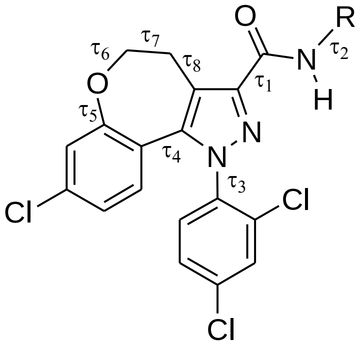

	E_{rel}	τ_1	τ_2	τ_3	τ_4	τ_5	τ_6	τ_7	τ_8
**1a-A**	0.00	-176	64	-74	-26	57	-95	55	-8
**1a-B**	0.23	-176	64	-73	-39	74	-40	-40	63
**1a-C**	1.40	-172	64	-72	-27	-4	51	-79	47
**1a-D**	0.63	-175	-121	-73	-27	57	-95	55	-8
**1b-A**	0.00	-175	65	-74	-26	57	-95	55	-8
**1b-D**	0.86	-177	-122	-74	-27	57	-95	55	-8
**1c-A**	0.00	-177	68	-74	-26	57	-95	55	-8
**1c-D**	0.83	-175	-127	-74	-27	57	-95	55	-8
**1d-A**	2.10	-176	63	-74	-26	57	-95	55	-8
**1d-D**	0.00	-178	-137	-74	-27	57	-95	55	-8
**1e-A**	0.00	-177	113	-74	-26	57	-95	55	-8
**1e-B**	0.10	-175	-87	-74	-26	57	-95	55	-8
**1e-C**	0.19	180	-118	-74	-26	57	-95	55	-8
**1e-D**	0.43	-176	90	-74	-26	57	-95	55	-8
**1f-A**	0.00	-178	180	-74	-27	57	-95	54	-7
